# Characterization of Calcium-Dependent Protein Kinase 2A, a Potential Drug Target Against Cryptosporidiosis

**DOI:** 10.3389/fmicb.2022.883674

**Published:** 2022-04-25

**Authors:** Fanfan Shu, Yu Li, Wenlun Chu, Xuehua Chen, Ziding Zhang, Yaqiong Guo, Yaoyu Feng, Lihua Xiao, Na Li

**Affiliations:** ^1^Center for Emerging and Zoonotic Diseases, College of Veterinary Medicine, South China Agricultural University, Guangzhou, China; ^2^State Key Laboratory of Agrobiotechnology, College of Biological Sciences, China Agricultural University, Beijing, China; ^3^Guangdong Laboratory for Lingnan Modern Agriculture, Guangzhou, China

**Keywords:** *Cryptosporidium parvum*, calcium-dependent protein kinase 2A, biologic function, inhibitor, enzyme, development

## Abstract

Calcium-dependent protein kinases (CDPKs) are important in calcium influx, triggering several biological processes in *Cryptosporidium* spp. As they are not present in mammals, CDPKs are considered promising drug targets. Recent studies have characterized *Cp*CDPK1, *Cp*CDPK3, *Cp*CDPK4, *Cp*CDPK5, *Cp*CDPK6, and *Cp*CDPK9, but the role of *Cp*CPK2A remains unclear. In this work, we expressed recombinant *Cp*CDPK2A encoded by the cgd2_1060 gene in *Escherichia coli* and characterized the biologic functions of *Cp*CDPK2A using qRT-PCR, immunofluorescence microscopy, immuno-electron microscopy, and *in vitro* neutralization. The results revealed that *Cp*CDPK2A protein was highly expressed in the apical region of sporozoites and merozoites and in macrogamonts. Monoclonal or polyclonal antibodies against *Cp*CDPK2A failed to block the invasion of host cells. Among the 44 candidate inhibitors from molecular docking of *Cp*CDPK2A, one inhibitor was identified as having a potential effect on both *Cryptosporidium parvum* growth and *Cp*CDPK2A enzyme activities. These data suggest that *Cp*CDPK2A may play some roles during the development of *C. parvum* and might be a potential drug target against cryptosporidiosis.

## Introduction

Human cryptosporidiosis is mainly caused by *Cryptosporidium hominis* and *Cryptosporidium parvum* (Checkley et al., 2015). The former is mainly transmitted anthroponotically among humans while the latter can be transmitted both anthroponotically among humans and zoonotically between farm animals and humans (Feng et al., 2018). Immunocompromised patients and infants under age two are especially susceptible to infections (Kotloff, 2017). Nitazoxanide, the only drug approved by the U.S. Food and Drug Administration, is ineffective in immunocompromised patients (Schneider et al., 2021). The development of anti-cryptosporidial drugs is hampered by poor understanding of the unique invasion process and energy metabolism of these parasites (Bhalchandra et al., 2018).

Calcium (Ca^2+^) is a ubiquitous intracellular signal controlling numerous cellular processes in apicomplexan parasites, including protein secretion, motility, and development, which are important in the invasion of host cells, egress of the pathogens from infected cells, gametogenesis, and other stages of the life cycle (Ghartey-Kwansah et al., 2020). In these pathogens, the calcium signal is received by higher hierarchical sensors such as calcium-dependent protein kinases (CDPKs; Wernimont et al., 2011). As CDPKs are absent in humans and animals, they are regarded as potential targets for chemotherapy (Magarinos et al., 2012). Thus far, several CDPKs have been identified in *C. parvum* by comparative genomics analysis (Lippuner et al., 2018).

In previous studies, *Cp*CDPK1, *Cp*DPK3, *Cp*CDPK4, *Cp*CDPK5, *Cp*CDPK6, and *Cp*CDPK9 proteins have been shown to play different roles in *C. parvum* biology. *Cp*CDPK1 is expressed in trophozoites and type I meronts of *C. parvum* (Choudhary et al., 2020). Inhibition of *Cp*CDPK1 expression had caused a reduction in parasite load *in vitro* (Castellanos-Gonzalez et al., 2016; Choudhary et al., 2020). Similarly, *Cp*CDPK1, *Cp*CDPK4, *Cp*CDPK6, and *Cp*CDPK9 have all been shown to participate in the invasion of host cells by *C. parvum* (Zhang et al., 2021; Su et al., 2022). In contrast, *Cp*CDPK3 and *Cp*CDPK5 might play some roles mostly during the growth of the parasite (Zhang et al., 2020).

In the present study, we have focused on *Cp*CDPK2A of *C. parvum* encoded by the cgd2_1060 gene. We examined its biochemical features and potential role in the development of *C. parvum*. One potential inhibitor of *Cp*CDPK2A was shown to reduce the growth of *C. parvum in vitro* at the micromolar level without cytotoxicity to the host cells.

## Materials and Methods

### Parasites and Host Cells

The IOWA strain of *C. parvum* was purchased from Waterborne, Inc. (New Orleans, United States) and stored at 4°C in PBS containing antibiotics for less than 3 months before use. Oocysts and free sporozoites were prepared as described previously (He et al., 2021). The human ileocecal adenocarcinoma cell line HCT-8 (ATCC CCL-244) was purchased from the Chinese Academy of Sciences for *in vitro* cultivation of *C. parvum* as described (Xu et al., 2019). Different intracellular stages of parasites were obtained by infecting HCT-8 cell for 24–48 h (Ni et al., 2020). Merozoites were collected from the medium of *C. parvum* cultures at 36 h post-infection by centrifugation (Zhang et al., 2015).

### Sequence Analysis of *Cp*CDPK2A

The entire open reading frame (ORF) of the cgd2_1060 gene was downloaded from the CryptoDB database.^1^ Function domains in the predicted amino acid sequence were searched using SMART (Simple Modular Architecture Research Tool; Letunic and Bork, 2018) and Motif Scan.^2^ The phylogenetic relationship of *Cp*CDPK2A to other *Cp*CDPKs was assessed using the neighbor-joining analysis implemented in Mega 6.0.^3^

### Expression and Purification of Recombinant *Cp*CDPK2A

The full-length cgd2_1060 gene of *C. parvum* was amplified using primers CDPK2A-F (CGGGATCCATGGGACAGGGCCAAAAC, with the *Bam*H I restriction site underlined) and CDPK2A-R (GCGTCGACAGAATTAGATCTTCTGAACATTTCC, with the *Sal* I restriction site underlined) and cloned into the pCold I vector. *Cp*CDPK2A was expressed in *Escherichia coli* BL21-CodonPlus (DE3)-RIPL component cells (Weidi Biotech, Shanghai, China). Following the initial overnight cultivation at 37°C on the solid Luria-Bertani (LB) medium containing 50 μg/ml ampicillin, the positive colonies were identified by PCR and cultured in the liquid LB medium containing 50 μg/ml ampicillin for 2–4 h until the OD_600_ reached 0.6–0.8. Afterward, the expression of the recombinant *Cp*CDPK2A (r*Cp*CDPK2A) was induced with 1 mM isopropyl-β-D-thiogalactopyranoside (IPTG) at 16°C for 24 h.

To purify r*Cp*CDPK2A, the culture was lysed by sonication. The r*Cp*CDPK2A in the lysate was purified using Ni-NTA His Bind Resin (Merck, Darmstadt, Germany), concentrated using the 30 kDa Amicon Ultra centrifugal filter Devices (Merck) at 5,000 *g* for 20 min, and eluted in PBS. The protein obtained was visualized using SDS-PAGE and analyzed for identity using the matrix-assisted laser desorption/ionization time of flight mass spectrometry (MALDI-TOF/MS; Sangon Biotech, Shanghai, China). The r*Cp*CDPK2A was stored at 4°C before use.

### Production of Polyclonal and Monoclonal Antibodies

Polyclonal antibodies were produced by GenScript Ltd. (Nanjing, China) using subcutaneously immunization with 0.4 mg purified r*Cp*CDPK2A dissolved in PBS buffer. Serum was harvested after five times immunization. Polyclonal IgG antibodies were purified using an affinity chromatographic method by GenScript Ltd. A monoclonal antibody against *Cp*CDPK2A was produced by AtaGenix Ltd. (Wuhan, China). Briefly, 8-week-old female BAB/c mice were immunized subcutaneously with 0.05 mg r*Cp*CDPK2A dissolved in PBS buffer. After three injections of r*Cp*CDPK2A, the spleen cells were fused with Sp2/0 cells using polyethylene glycol 1,500 (Sigma-Aldrich, Missouri, United States). Positive hybridomas were selected by testing the hybridoma supernatants using ELISA plates coated with 0.2 μg/well r*Cp*CDPK2A. After two rounds of subcloning by single-cell limiting dilution to ensure clonality, the monoclonal antibody was purified using Protein A + G Agarose (Beyotime, Shanghai, China) and stored at −80°C before use.

### Analysis of Recombinant and Native *Cp*CDPK2A

The r*Cp*CDPK2A protein was examined using Western blot analysis. Briefly, the protein was electrophoresed on 10% SDS-PAGE and transferred into a polyvinylidene fluoride (PVDF) membrane (Millipore, Billerica, United States). The membrane was probed with anti-His-tag antibodies (Cell Signaling Technology, Danvers, United States) at 1:1,000 dilution as the primary antibody and peroxidase-conjugated goat anti-mouse IgG (Beyotime) at 1:500 dilution as the secondary antibody. The reactivity was visualized using an enhanced High-sig ECL Western Blotting Substrate (Tanon, Shanghai, China).

For Western blot analysis of native *Cp*CDPK2A, 8 × 10^7^ sporozoites of *C. parvum* were freeze-thawed between −80°C and 4°C in sterile water containing 1% protease inhibitor cocktail (Sigma-Aldrich). The lysates and purified r*Cp*CDPK2A were electrophoresed using 10% SDS-PAGE, transferred into PVDF membranes (Millipore), and incubated with anti-*Cp*CDPK2A polyclonal serum (1:200 dilution) or control serum (1:200 dilution), and incubated with anti-*Cp*CDPK2A monoclonal antibody (1:200 dilution) or purified IgG from native mouse serum (1:200 dilution). The bands in Western blots were developed as described above.

### Assessment of Expression of *Cp*CDPK2A Gene

The relative expression of the *Cp*CDPK2A gene in developmental stages of *C. parvum* in HCT-8 cells was evaluated using qPCR. Total RNA was isolated from *C. parvum* cultured in HCT-8 cells for 2, 6, 12, 24, 36, 48, and 72 h using RNeasy Mini Kit (Qiagen, Dusseldorf, Germany). cDNA was generated by the GoScript™ Reverse Transcription System (Promega, Wisconsin, United States) using random primers. The relative concentration of the *Cp*CDPK2A gene transcript was measured using a SYBR1 Green-based qPCR on a Lightcycler 480 Instrument II (Roche, Basel, Switzerland) and the following primers: (TAATGGGTTCGAGAAGAAATGG) and (TGAGCTATGACAGTAAGGG CAA; amplicon size = 163 bp). Threshold cycle (*C*_T_) values of the 18S rRNA gene of *C. parvum* were used in data normalization as described (Mauzy et al., 2012). The relative expression levels of the *Cp*CDPK2A gene were calculated using the delta–delta method (Livak and Schmittgen, 2001). All measurements were performed in triplicate.

### Characterization of *Cp*CDPK2 Expression in *Cryptosporidium parvum*

For the examinations of *Cp*CDPK2A expression using immunofluorescence assay (IFA), parasites were air-dried on SuperStick coverslips (Waterborne), fixed in 4% paraformaldehyde for 15 min, permeabilized and blocked in PBS containing 0.1% Triton-100 and 5% BSA, and labeled with anti-*Cp*CDPK2A polyclonal serum (1:200 dilution). Alexa Fluor 594-conjugated anti-rabbit IgG (H + L; Cell Signaling Technology) was used as the secondary antibody in IFA. The cells were counter-stained with the 4′, 6-diamidino-2-phenylindole (DAPI; Beyotime). Epifluorescence images were captured on an Olympus BX53 fluorescence microscope (Olympus, Tokyo, Japan).

For immuno-electron microscopy (IEM) of oocysts, sporozoites, and cultured parasites, samples were fixed in 4% paraformaldehyde and 0.1% glutaraldehyde in PBS overnight at 4°C, washed with PBS buffer, and embedded in low melting agarose. After dehydration in a gradient ethanol series at −20°C, the samples were infiltrated with LR White acrylic resin (Sigma) overnight at −20°C and polymerized with fresh LR White in gelatin capsules at −25°C for 3 days. Thin slices (50 nm in thickness) were sectioned using a diamond knife (Leica, Welzlar, Germany) and mounted on nickel grids. After three washes, the sections were blocked with 1% BSA and incubated with affinity-purified anti-*Cp*CDPK2A polyclonal antibodies (1:200 dilution) followed by secondary anti-rabbit IgG conjugated to 10 nm colloidal gold (1:200 dilution; Sigma). After counter-staining with uranyl acetate and Reynold’s lead citrate, the sections were examined under a Talos L120C transmission electron microscope (Thermo Fisher, Waltham, United States). Blank controls with no primary antibodies were included in the IEM examination to exclude the occurrence of non-specific binding of gold-conjugated antibodies.

### *In vitro* Neutralization of Invasion

A neutralization assay was performed to evaluate the effect of antibodies against *Cp*CDPK2A on *C. parvum* infection in HCT-8 cells. Host cells were cultured on coverslips in 24-well plates until 90% confluence. The culture was inoculated with bleach-treated oocysts in the presence of anti-*Cp*CDPK2A polyclonal serum (dilution 1:1000, 1:500, and 1:200) or anti-*Cp*CDPK2A monoclonal antibody (1, 10, and 20 μg/ml) at 37°C for 15 min. After 2 h infection, unexcysted oocysts were removed by washing with culture medium. Monolayers were cultured for 24 h and washed three times with PBS. The coverslips were fixed, permeabilized, and blocked as described before. Intracellular *C. parvum* stages were labeled with Cy3-labeledpolyclonal anti-*C. parvum* antibody Sporo-Glo (Waterborne). Fifty random images were taken under immunofluorescence microscopy and the parasites in them were counted using ImageJ.^4^ The neutralization effects were calculated as previously described (Upton et al., 1995). All assays were performed in triplicate and data from different groups were compared using the Student’s *t*-test.

### Inhibitory Effect of *Cryptosporidium parvum* Invasion and Growth With Candidate Inhibitors of *Cp*CDPK2A

Using molecular docking of the simulated *Cp*CDPK2A structure, small molecules were selected from the ChemDiv database. The binding energy values of ligands to proteins were calculated based on ligand efficiency, Coulomb energy, Van der waals energy, and H-bond energy. Candidate small molecules were purchased from TopScience Ltd. (Shanghai, China) and screened for activities against *C. parvum* development *in vitro* using qRT-PCR (Zhang et al., 2012). Briefly, HCT-8 monolayers (2 × 10^4^ cells/well) grown in 96 well plates were inoculated with hypochlorite-treated oocysts (2 × 10^4^ oocysts/well) containing dilutions of compounds (10 μM) or DMSO (1%). The parasites were allowed to invade host cells for 3 h, and uninvaded parasites were washed off with the medium change. The cultures containing newly added compounds were incubated for an additional 41 h for the isolation of total RNA using RNeasy Mini Kit (Qiagen). The levels of 18S rRNA transcripts of *C. parvum* and HCT-8 cells were measured by using the HiScript II One Step qRT-PCR SYBR Green kit (Vazyme Biotech, Nanjing, China) as described (Zhang et al., 2012). The parasite loads in cultures were calculated using the ΔΔ*C*_T_ method (Cai et al., 2005). Compounds showing >60% inhibition at 10 μM in the first screening were selected for further assessment and measurement of their half-maximal effective concentration (EC_50_) using dilutions ranging from 10 nM to 20 μM in DMSO. The cytotoxicity of compounds was evaluated by the Cell Titer 96 Aqueous One Solution Cell Proliferation Assay (Promega) as described (Pobsuk et al., 2019). The half-maximal toxic concentration (TC_50_) values on host cells were calculated using Graphpad Prism 7 (Graphpad software, San Diego, United States).

Candidate anti-cryptosporidial compounds were further examined for their effects on *C. parvum* invasion or development assays. In invasion experiments, HCT-8 cells plated on coverslips were infected with bleached oocysts and cultured with active compounds for 3 h at 37°C. In growth experiments, monolayers were infected with bleached oocysts, washed twice with PBS at 3 h post-infection, and returned to culture in fresh HCT-8 medium with active compounds for 41 h. RNA was collected from each well at specified time point to assess the anti-cryptosporidial activities of these compounds using qRT-PCR method described above. The addition of 140 μM paromomycin was used as a positive control. Parasite enumeration by immunofluorescence microscopy was used to verify the efficacy of active compound K292-0423 against *C. parvum* development *in vitro*.

### Measurement of Enzyme Activities of r*Cp*CDPK2A

The enzymatic activity of r*Cp*CDPK2A was measured using the ELISA-based NADH-coupled ATPase assay as described previously (Rutaganira et al., 2017). The kinase reaction was conducted at 30°C for 40 min in 100 mM HEPES, pH 8.0, 150 mM NaCl, 10 mM MgCl_2_, 1 mM CaCl_2_, 50 mM KCl, 10 mM DTT, 2 μg/ml BSA, and 0.01% Tween 20. The typical assay of 100 μl reactions contained 50 μM Syntide-2, 50 μM ATP, 150 μM NADH, 300 μM phosphoenolpyruvate (PEP), and a mixture of pyruvate kinase (4 units/ml) and lactate dehydrogenase (6 units/ml; Sigma). The reaction was initiated with the addition of 100 nM r*Cp*CDPK2A. A recombinant insulinase-like protease of *C. parvum*, INS-16, was used as the negative control. To assess the inhibitory effect on enzymatic activities of r*Cp*CDPK2A, six active compounds were added to the reaction at 10 μM in duplicate. Compounds with significant inhibition rates were selected to determine the half-maximal inhibition concentration (IC_50_) using final concentrations of 1 μM to 20 μM. The IC_50_ values were calculated using Graphpad Prism 7.

## Results

### Sequence Characteristics of *Cp*CDPK2A

**Figure 1:**
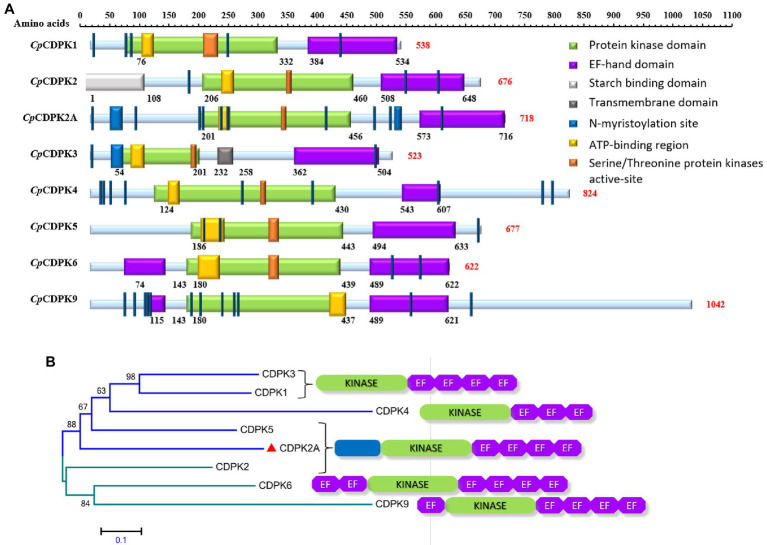
Domain structures and phylogenetic relationships of calcium-dependent protein kinases in *Cryptosporidium parvum* (*Cp*CDPKs). **(A)** Predicted domain structures of *Cp*CDPK2A and other *Cp*CDPKs using SMART and Motif Scan. **(B)** Phylogenetic relationships of *Cp*CDPK2A (indicated with a red triangle) to other *Cp*CDPKs based on neighbor-joining (NJ) analysis of the amino acid sequences, with the number and location of kinase and EF-hand domains being indicated.

*Cp*CDPK2A encoded by the cgd2_1060 gene contains 718 amino acids without a predicted signal peptide and transmembrane domain. It has a typical serine/threonine kinase domain and four EF-hand motifs, one ATP-binding region, and 12 N-myristoylation sites (Figure 1A). In phylogenetic analysis of amino acid sequences, *Cp*CDPK2A is most related to *Cp*CDPK5 (Figure 1B).

### Production of r*Cp*CDPK2A in *Escherichia coli*

**Figure 2:**
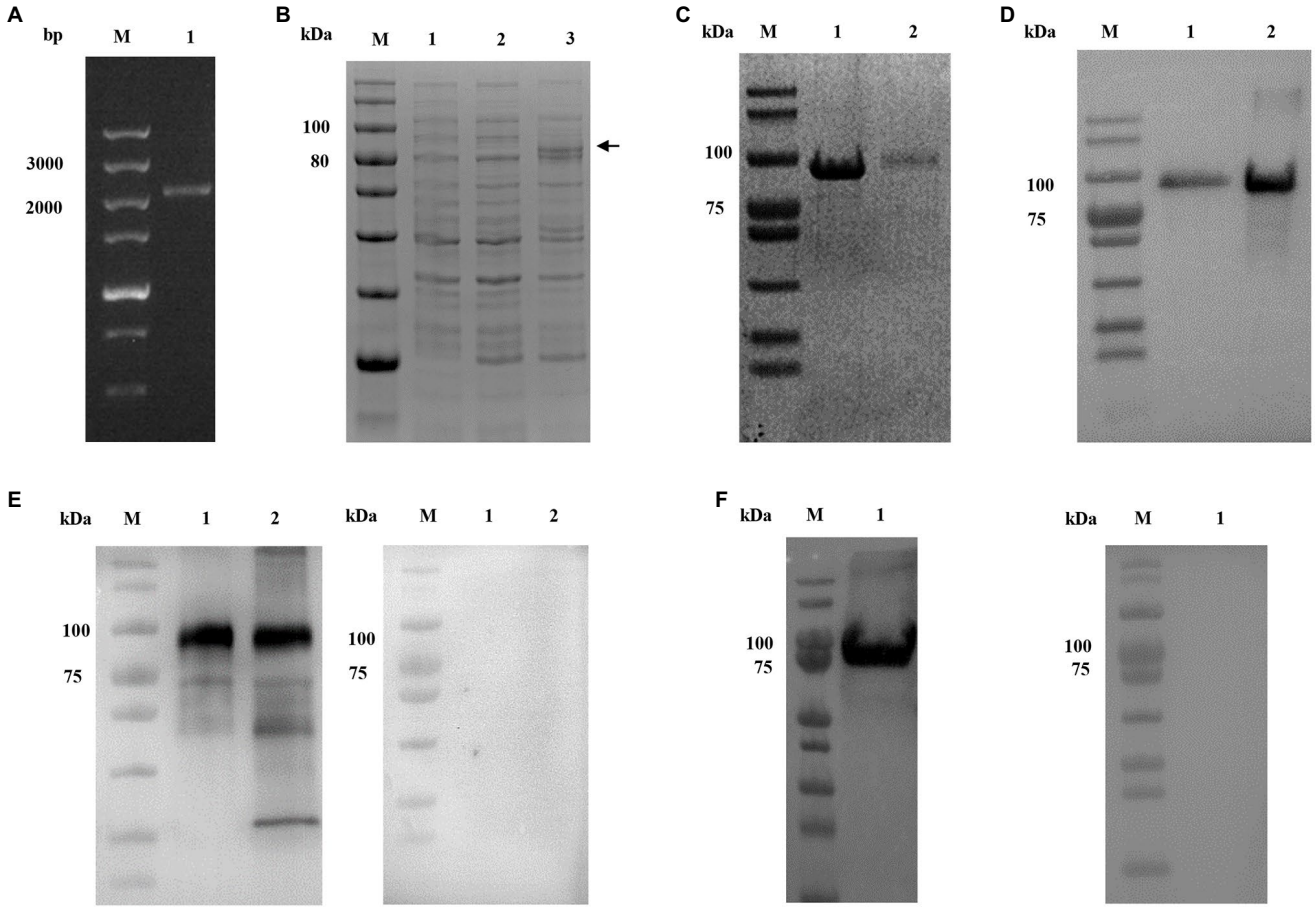
Production and characterization of r*Cp*CDPK2A of *Cryptosporidium parvum*. **(A)** PCR amplification of the cgd2_1060 gene of *C. parvum*. Lane M: molecular markers; Lane 1: cgd2_1060 PCR product. **(B)** Expression of r*Cp*CDPK2A in *Escherichia coli* BL21-CodonPlus (DE3)-RIPL. Lane M: protein size markers; Lane 1: bacterial lysate of pCold I vector control; Lane 2: bacterial lysate transformed with the recombinant plasmid without IPTG induction; and Lane 3: bacterial lysate transformed with the recombinant plasmid with IPTG induction for 24 h, with the expected product being indicated by a black arrow. **(C)** Purified r*Cp*CDPK2A from the Ni-NAT affinity column. Lane M: protein size markers; Lane 1: r*Cp*CDPK2A protein purified from the inclusion body; and Lane 2: r*Cp*CDPK2A protein purified from culture supernatant. **(D)** Western blot analysis of purified r*Cp*CDPK2A using anti-His-tag monoclonal antibody. Lane M: protein size markers; Lane 1: r*Cp*CDPK2A protein purified from culture supernatant; and Lane 2: r*Cp*CDPK2A protein purified from the inclusion body. **(E)** Western blot analysis of native *Cp*CDPK2A in sporozoites of *C. parvum* with post-immune serum (left panel) and control serum (right panel). Lane M: protein size markers; Lane 1: r*Cp*CDPK2A protein. Lane 2: crude proteins extracted from sporozoites. **(F)** Western blot analysis of purified r*Cp*CDPK2A using anti-*Cp*CDPK2A monoclonal antibody (left panel) and control antibody (right panel).

The full-length of *Cp*CDPK2A (2,151 bp) gene was amplified from *C. parvum* genomic DNA by PCR (Figure 2A). The r*Cp*CDPK2A with a N-terminal His-tag was expressed in *E. coli* BL21-CodonPlus (DE3)-RIPL (Figure 2B) and purified from the supernatant with a single band of about ~80 kDa (Figure 2C) and confirmed by Western blot analysis using the anti-his monoclonal antibody (Figure 2D). The identity of the r*Cp*CDPK2A generated was further confirmed by MALDI-TOF/MS analysis (data not shown).

The purified r*Cp*CDPK2A was used in the generation of antibodies against *Cp*CDPK2A. Anti-*Cp*CDPK2A polyclonal serum produced recognized one single band on the r*Cp*CDPK2A and the native proteins of *C. parvum* sporozoites, but no reaction was detected on the negative control group (Figure 2E). Similarly, anti-*Cp*CDPK2A monoclonal antibody also recognized one single band of r*Cp*CDPK2A, but no reaction was detected on the negative control group (Figure 2F).

### Expression of the *Cp*CDPK2A Gene in *Cryptosporidium parvum* Culture

**Figure 3:**
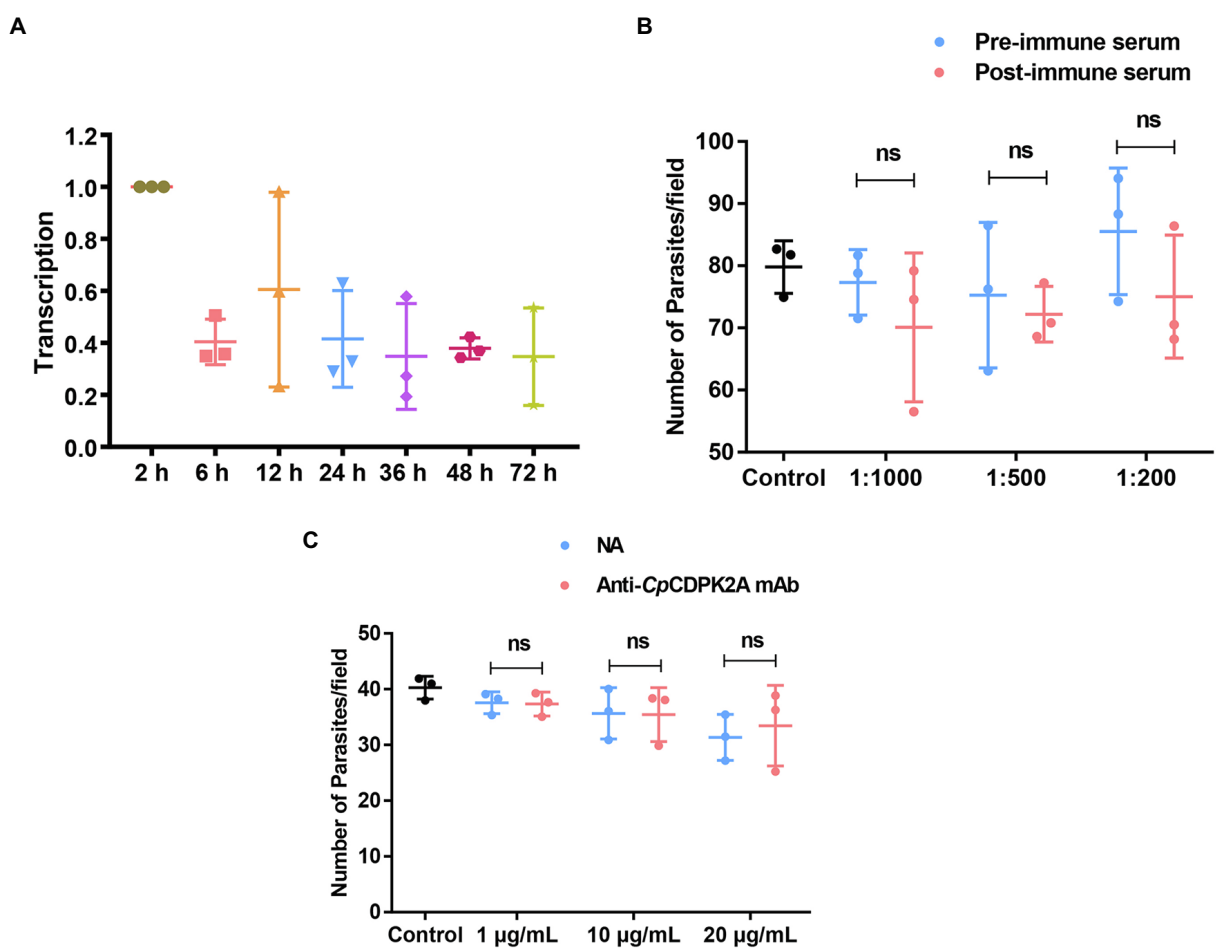
Transcription profile of the gene encoding *Cp*CDPK2A and neutralization efficiency of antibodies against the recombinant protein. **(A)** Relative levels of the mRNA expression of the gene in different developmental stages of *Cryptosporidium parvum*. **(B)** Neutralization efficiency of sporozoite invasion *in vitro* by polyclonal serum against r*Cp*CDPK2A. **(C)** Neutralization efficiency of sporozoite invasion *in vitro* by anti-*Cp*CDPK2A monoclonal antibody. Anti-*Cp*CDPK2A mAb: IgG purified from hybridoma supernatants; NA: IgG purified from native mouse serum. Error bars are the standard error of mean from three independent assays.

The expression of the cgd2_1060 gene encoding *Cp*CDPK2A during the *in vitro* development of *C. parvum* was followed by qRT-PCR. The result showed reasonable expression of the gene over the 72-h infection course of HCT-8 cells. The peak expression occurred at 2 and 12 h post-infection (Figure 3A).

### Limited Neutralization of *Cryptosporidium parvum* Invasion

Poor inhibitory effects were achieved in the assessment of the neutralization of *C. parvum* infection by both anti-*Cp*CDPK2A polyclonal serum (Figure 3B) and anti-*Cp*CDPK2A monoclonal antibody (Figure 3C). The inhibitory efficacy of polyclonal serum on *C. parvum* infection of HCT-8 cells was 8.2% (77.3 ± 4.2 and 70.9 ± 9.7 for pre- and post-immune serum, respectively; *t*_(2)_ = 0.940, *p* = 0.446) at 1:1,000 dilution, 4.3% (75.2 ± 9.5 and 71.9 ± 3.3 for pre- and post-immune serum, respectively; *t*_(2)_ = 0.598, *p* = 0.610) at 1:500 dilution, and 9.3% (82.7 ± 6.4 and 75.0 ± 8.0 for pre- and post-immune serum, respectively; *t*_(2)_ = 2.467, *p* = 0.132) at 1:200 dilution (the number of parasites in the control group was 79.7 ± 3.4; Figure 3B). The inhibitory efficacy of anti-*Cp*CDPK2A monoclonal antibody was 0.5% (37.5 ± 1.6 and 37.3 ± 1.7 for control antibody and monoclonal antibody, respectively; *t*_(2)_ = 1.031, *p* = 0.411) at 1 μg/ml, 0.6% (35.6 ± 3.7 and 35.4 ± 3.9 for control antibody and monoclonal antibody, respectively; *t*_(2)_ = 0.791, *p* = 0.512) at 10 μg/ml, and −6.7% (31.3 ± 3.3 and 33.4 ± 5.9 for control antibody and monoclonal antibody, respectively; *t*_(2)_ = 0.123, *p* = 0.913) at 20 μg/ml (the number of parasites in the control group was 40.3 ± 1.7; Figure 3C).

### Expression Pattern of *Cp*CDPK2A in Developmental Stages of *Cryptosporidium parvum*

**Figure 4:**
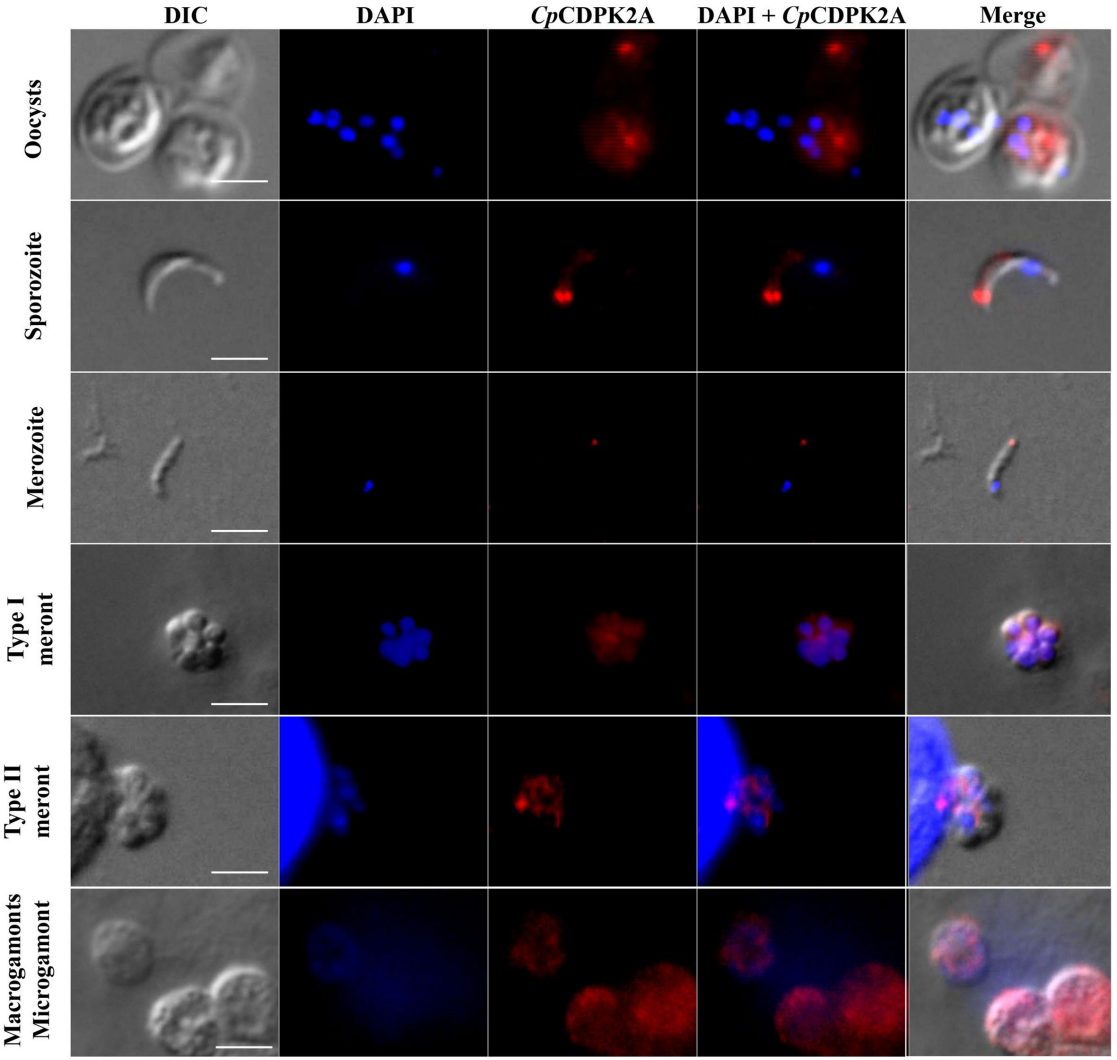
Localization of the expression of native *Cp*CDPK2A in *Cryptosporidium parvum* by immunofluorescence microscopy. The expression of *Cp*CDPK2A in oocysts (first panel), a sporozoite (second panel), a merozoite (third panel), a type I meront (fourth panel), a type II meront (fifth panel), a microgamont, and macrogamonts (sixth panel) are shown (in red). Nuclei were counter-stained with DAPI (in blue). Bars = 2 μM.

In immunofluorescence microscopy, anti-*Cp*CDPK2A polyclonal antibodies reacted with the content of oocysts in a dotted pattern (Figure 4 first panel). In free sporozoites, the fluorescence signal was observed mostly around the apical region with two bright dots (Figure 4 second panel). In merozoites, the fluorescence was at the apical region with one dot (Figure 4 third panel). The antibodies also reacted with contents rather than parasitophorous vacuole membrane (PVM) of type I and type II meronts (Figure 4 fourth and fifth panels). Between microgamonts and macrogamonts, the antibodies stained the latter more intensively (Figure 4 sixth panel).

**Figure 5:**
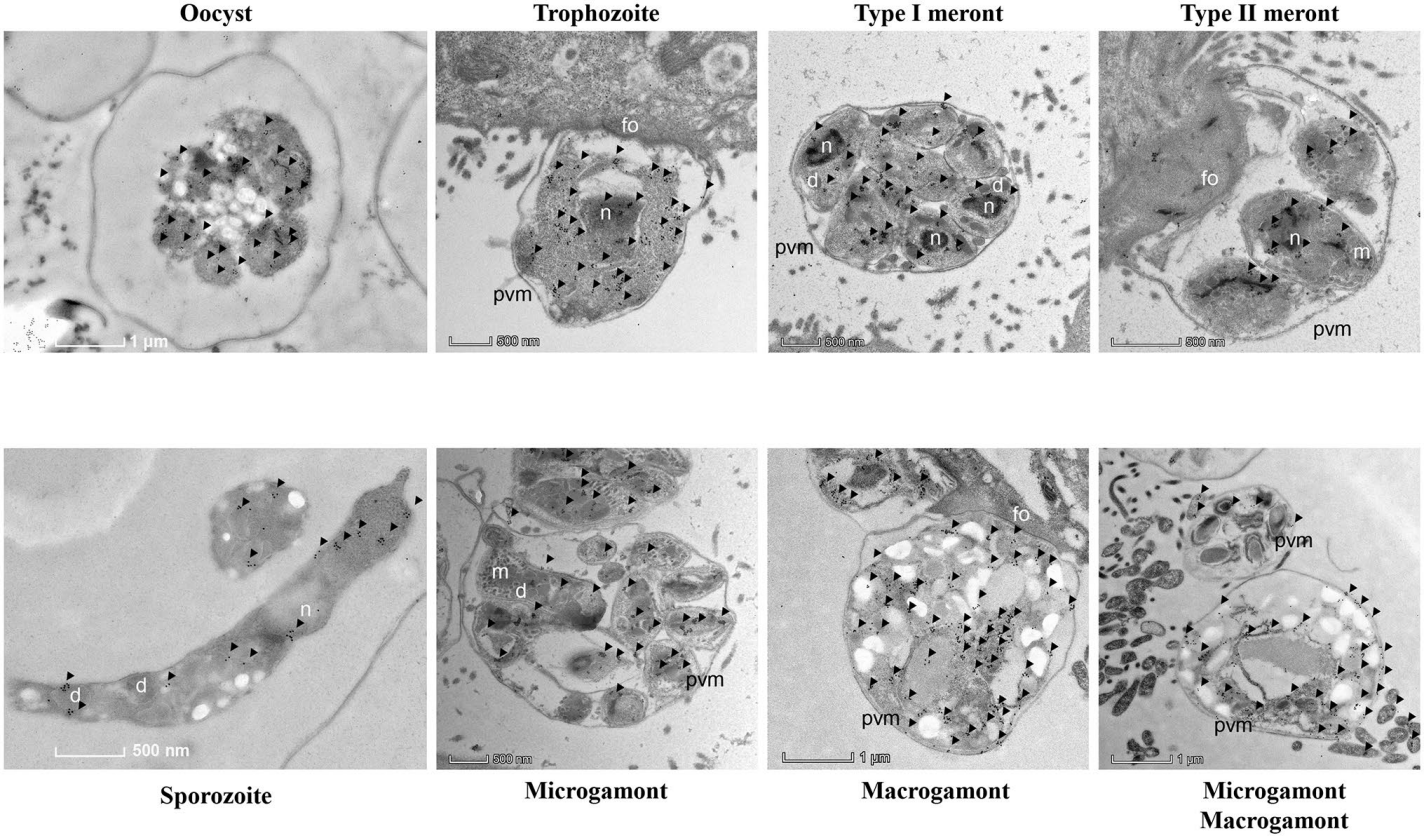
Subcellular distribution of *Cp*CDPK2A protein in *Cryptosporidium parvum* by immuno-electron microscopy. Expression of *Cp*CDPK2A in an oocyst, a sporozoite, a trophozoite, a type I meronts, a type II meronts, microgamonts, and macrogamonts. No gold particles were observed on the micronemes (m), parasitophorous vacuole membrane (pvm), and feeder organelles (fo), with a few gold particles seen on dense granules (d). Gold particles are indicated by black arrows.

The results of IEM analysis confirmed the distribution of *Cp*CDPK2A in developmental stages of *C. parvum*. In IEM analysis of oocysts, the colloidal-gold particles were mostly on sporozoites (Figure 5). In free sporozoites, although gold particles were detected in other areas of the body in a dotted pattern, they had the most accumulation in two sides near the apical end (Figure 5). In IEM analysis of developmental stages in culture, gold particle accumulations were more intense in trophozoites, type I meronts, and macrogamonts than in type II meronts and microgamonts (Figure 5).

### Inhibitory Effect of Potential Inhibitors of *Cp*CDPK2A Against the Development of *Cryptosporidium parvum in vitro*

**Figure 6:**
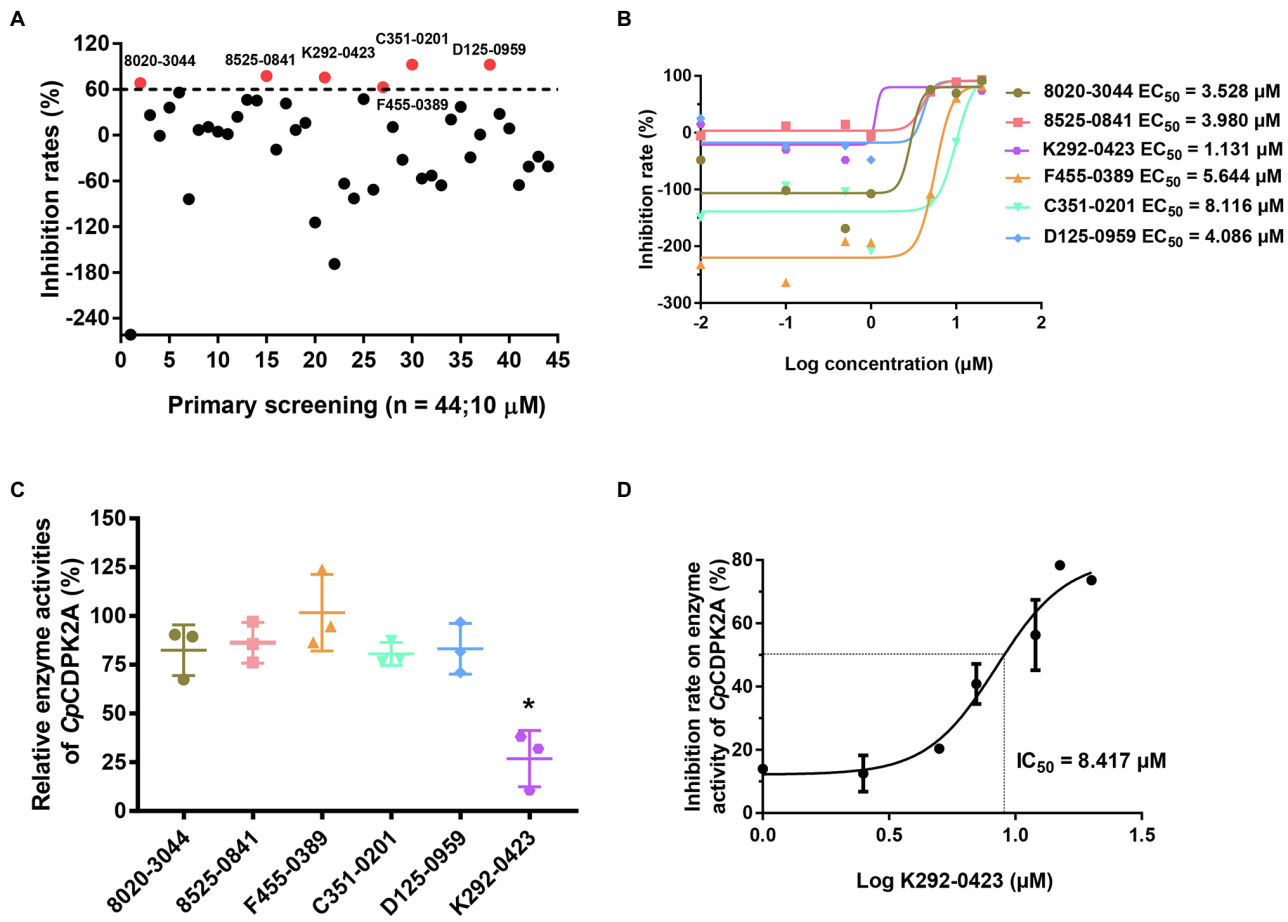
Inhibition of *Cryptosporidium parvum* growth in *in vitro* HCT-8 cell cultures by candidate *Cp*CDPK2A inhibitors from molecular docking. **(A)** Efficacy of 44 compounds in primary screening. Six compounds displayed potential efficacy (>60%) are indicated by red dots and names. Data are the means from two independent assays. **(B)** Half-maximal effective concentrations (EC_50_) of the six active compounds. Data are the mean ± SD from two independent assays, with those from the control group treated with DMSO being used in data normalization. **(C)** Relative enzyme activities of *Cp*CDPK2A after incubation with the six active compounds. Only K292-0423 displayed significant inhibition of *Cp*CDPK2A (*p* = 0.0128). *P < 0.05. Error bars are the SEM from two independent assays. **(D)** Half-maximal inhibition concentration (IC_50_) of K292-0423 on *Cp*CDPK2A in a dose-response experiment. Data are the mean ± SD from two independent assays.

A total of 50 compounds were selected from the ChemDiv core database according to the scores from molecular docking of *Cp*CDPK2A (Table 1). In primary screening at 10 μM, a wide range of anti-parasite inhibition rates of these compounds on *C. parvum* development were obtained, ranging from −261.50 to 92.55% (Figure 6A). The negative values were indicative of the cytotoxicity of some of the compounds. Among them, six compounds (8020-3044, 8525-0841, K292-0423, F455-0389, C351-0201, and D125-0959) were able to inhibit the parasite growth by >60%. Subsequent dose–response evaluations of them reveal that the anti-cryptosporidial EC_50_ values ranged from 1.13 to 8.11 μM (Figure 6B). The cytotoxicity of these six top hits was evaluated in HCT-8 cells using a cell proliferation assay (Table 2). Three top hits showed minor cytotoxicity on the growth of HCT-8 cells (TC_50_ = 15.30 μM for 8020-3044, TC_50_ = 41.16 μM for F455-0389, and TC_50_ = 10.73 μM for D125-0959). The remaining three top hits displayed no mammalian cytotoxicity, with TC_50_ scores of >75 μM (Table 2).

**Table 1:** Scores of 50 top small compounds from molecular docking of *Cp*CDPK2A.

No.	Name	Glide score	Ligand efficiency (kcal/mol)	Coulomb energy (kcal/mol)	Van der waals energy (kcal/mol)	H-bond energy (kcal/mol)
1	F019-4325	−7.83	−0.37	−6.79	−31.42	−0.30
2	8020-3044	−7.70	−0.23	−12.97	−32.41	−0.55
3	D074-0339	−7.66	−0.28	−20.22	−21.93	−0.38
4	5516-0721	−7.58	−0.26	−21.26	−32.58	−0.91
5	Y020-8606	−7.47	−0.34	−5.33	−28.23	−0.48
6	D463-0173	−7.46	−0.25	−9.45	−34.60	−0.25
7	G857-1048	−7.39	−0.26	−9.61	−35.89	−1.18
8	D126-0031	−7.36	−0.27	−14.66	−30.17	−0.55
9	7999-4509	−7.30	−0.36	−12.18	−24.41	−0.93
10	8020-0786	−7.22	−0.30	−13.44	−25.55	−0.93
11	Y020-4510	−7.17	−0.29	−2.87	−35.20	−0.18
12	N094-0017	−7.13	−0.48	−3.99	−23.61	−0.54
13	D718-0232	−7.11	−0.25	−6.18	−37.91	−0.30
14	Y080-1179	−7.10	−0.34	−6.54	−30.04	−0.61
15	6191-0635	−7.09	−0.32	−17.36	−17.44	−0.94
16	D174-0560	−7.07	−0.24	−15.46	−30.39	−0.44
17	8525-0841	−7.00	−0.26	−8.87	−32.26	−0.46
18	D503-0028	−6.98	−0.35	−2.01	−30.57	−0.04
19	Y041-4264	−6.98	−0.32	−2.35	−33.82	−0.24
20	D724-0710	−6.94	−0.25	−4.49	−35.37	−0.07
21	J106-0269	−6.92	−0.23	−4.12	−36.24	−0.29
22	D718-0261	−6.91	−0.35	−8.63	−25.90	−0.71
23	K292-0423	−6.90	−0.25	−7.29	−40.47	−0.88
24	8009-8646	−6.90	−0.29	−4.87	−30.36	−0.61
25	D718-1386	−6.88	−0.33	−14.76	−22.57	−0.43
26	S214-0240	−6.87	−0.26	−13.33	−22.82	−0.70
27	D361-0309	−6.86	−0.25	−5.11	−34.34	−0.10
28	G540-0685	−6.86	−0.27	−4.48	−38.59	−0.32
29	D245-0084	−6.82	−0.28	−6.71	−33.20	−0.21
30	4094-0127	−6.81	−0.31	−6.41	−29.93	−0.61
31	F455-0389	−6.79	−0.22	−8.73	−38.25	−0.26
32	S641-0954	−6.79	−0.30	−15.37	−24.74	−0.51
33	D338-0003	−6.79	−0.20	−10.39	−40.64	−0.46
34	C351-0201	−6.78	−0.31	−2.16	−32.28	−0.23
35	8020-3749	−6.75	−0.25	−15.83	−30.79	−0.16
36	T835-1404	−6.73	−0.31	−6.93	−26.44	−0.61
37	6321-0317	−6.70	−0.28	−5.54	−33.71	−0.35
38	C630-0317	−6.69	−0.22	−4.63	−35.49	−0.29
39	D114-0037	−6.68	−0.28	−6.47	−30.63	−0.42
40	M333-0427	−6.67	−0.27	−13.83	−29.77	−0.16
41	FF01-7800	−6.66	−0.48	−3.75	−19.50	−0.49
42	F092-0379	−6.64	−0.24	−8.11	−32.51	−0.46
43	C200-5998	−6.64	−0.28	−2.09	−32.14	−0.22
44	D125-0959	−6.63	−0.23	−2.04	−35.51	−0.29
45	K832-2906	−6.62	−0.21	−2.54	−40.10	−0.08
46	4327-3149	−6.62	−0.41	−9.38	−23.03	−0.30
47	D585-0060	−6.53	−0.24	−14.51	−25.47	−0.49
48	M191-0009	−6.52	−0.28	−13.64	−26.56	−0.88
49	P930-1365	−6.51	−0.26	−12.26	−27.70	−0.46
50	P207-8877	−6.51	−0.43	−3.08	−21.60	−0.59

**Table 2:** Half-maximal effective concentrations (EC_50_) and half-maximal toxic concentrations (TC_50_) of six candidate *Cp*CDPK2A inhibitors in HCT-8 cultures of *Cryptosporidium parvum.*

No.	Name	EC_50_ (**μ**M)	TC_50_ (**μ**M)	SI*
2	8020-3044	3.528	15.30	4.33
17	8525-0841	3.980	>75	>18.84
23	K292-0423	1.131	>75	>66.31
31	F455-0389	5.644	41.16	7.29
34	C351-0201	8.116	>100	>12.32
44	D125-0959	4.086	10.73	2.62

*SI: safety index.

### Inhibition of r*Cp*CDPK2A Enzyme Activity

The six candidate *Cp*CDPK2A inhibitors were assessed for their inhibitory effects on the enzymatic activity of r*Cp*CDPK2A using the NADH-coupled enzyme assay. In pre-experiments, the catalytic efficiency of r*Cp*CDPK2A was 94.3 ± 11.6 nmol/mg/min, with Kcat of 76.0 min^−1^ at 30°C, pH = 7.5 and Michaelis constant (K_m-Syntide-2_) of 66.6 ± 21.2 μM. Among the six candidate inhibitors, only K292-0423 inhibited the activity of r*Cp*CDPK2A at 10 μM (inhibition rate = 73.1%, *p* = 0.0009, *t* = 8.753; Figure 6C). The IC_50_ of K292-0423 on the enzyme was 8.417 μM (Figure 6D).

**Figure 7:**
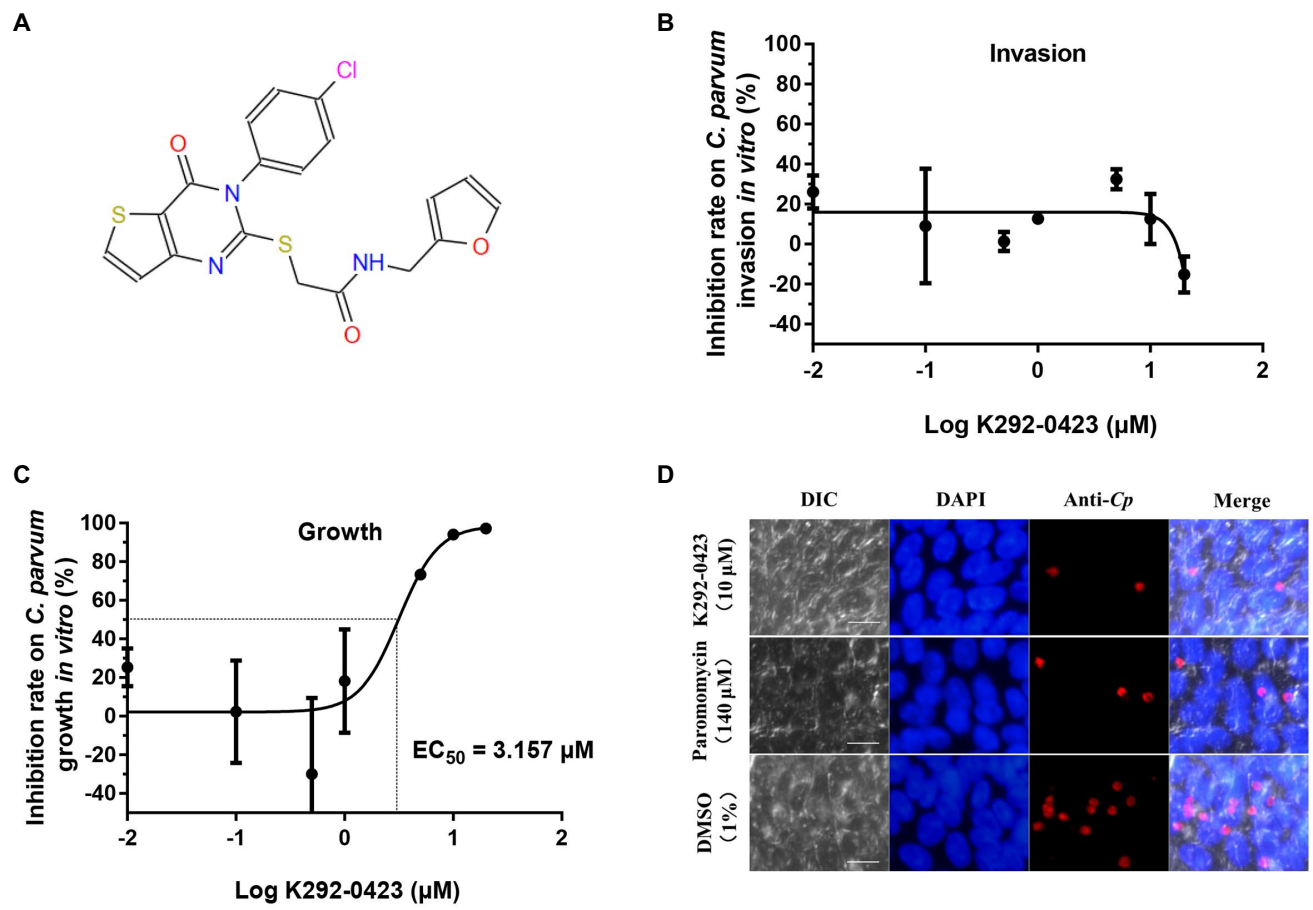
Effects of *Cp*CDPK2A inhibitor K292-0423 on the invasion and growth of *Cryptosporidium parvum*. **(A)** Structure of K292-0423. **(B)** Effect of K292-0423 on the *C. parvum* invasion using RT-qPCR analysis of cultures treated with the compound only during the invasion phase. Data are the mean ± SD from two independent assays. **(C)** Effect of K292-0423 *C. parvum* invasion using RT-qPCR analysis of cultures treated with the compound after the sporozoite invasion. Data are the mean ± SD from two independent assays. **(D)** Representative images of *C. parvum* cultures treated with K292-0423 (10 μM), paromomycin (140 μM), or DMSO control (1%). The developmental stages were stained red with anti-*Cryptosporidium* antibodies while the host cell nuclei stained blue with 4′,6-diamidino-2-phenylindole (DAPI). Scale = 10 μM.

In further evaluations of the K292-0423 (Figure 7A), the compound had poor effects against the invasion of HCT-8 cells by *C. parvum* (inhibition rates = −24.27 to 37.66% at 0.01 to 20 μM; Figure 7B). In contrast, significant inhibitory effects of K292-0423 on parasite loads were observed after the compound was added to the culture after the invasion of cells by sporozoites, with an EC_50_ of 3.157 μM (Figure 7C). This was confirmed by direct enumeration of parasites in cultures treated with K292-0423, which at 10 μM generated inhibitory effects similar to that by the positive control paromomycin (Figure 7D).

## Discussion

The results of the present study suggest that *Cp*CDPK2A might play an important role in the growth of *C. parvum*. This canonical CDPK is expressed in multiple developmental stages of the pathogen, especially sporozoites, trophozoites, type I meronts, and macrogamonts, suggesting that *Cp*CDPK2A potentially plays multiple roles in the development of *C. parvum*. This is supported by results of *in vitro* studies with candidate inhibitors of the enzyme from molecular docking, which produced significant inhibitory effects on *Cp*CDPK2A and *C. parvum* growth by one inhibitor. Thus, like *Cp*CDPK1, *Cp*CDPK2A can potentially serve as a target for the development of therapeutic agents against cryptosporidiosis.

*Cp*CDPK2A is one of the CDPK genes highly expressed in multiple life cycle stages of *C. parvum*. Previously, based on RNAseq analysis, *Cp*CDPK1 and *Cp*CDPK6 appeared to be more abundantly expressed in life cycle stages of *C. parvum* than other *Cp*CDPKs (Lippuner et al., 2018). Data from the present study suggest that *Cp*CDPK2A is another CDPK that is expressed in multiple life cycle stages of *C. parvum*, including sporozoites, trophozoites, type 1 meronts, and macrogamonts. However, data obtained from studies of *Cp*CDPK1, *Cp*CDPK2A, *Cp*CDPK3, *Cp*CDPK4, *Cp*CDPK5, *Cp*CDPK6, and *Cp*CDPK9 indicate that these CDPKs are all expressed in sporozoites and asexual stages of *C. parvum*, although the relative levels of them could be different (Zhang et al., 2020, 2021; Su et al., 2022). Their subcellular locations, however, appear to differ among *Cp*CDPKs. For example, in sporozoites, *Cp*CDPK1, *Cp*CDPK3, and *Cp*CDPK5 are expressed over the entire body, *Cp*CDPK2A, *Cp*CDPK4, and *Cp*CDPK9 are expressed at the apical end, while *Cp*CDPK6 is expressed in what appeared to be dense granules (Zhang et al., 2021). The particular apical pattern of the *Cp*CDPK2A expression in sporozoites and merozoites makes it very unusual among *Cp*CDPKs.

Despite the apical location of its expression in sporozoites and merozoites, *Cp*CDPK2A does not appear to be involved directly in the invasion process of *C. parvum*. At the transcription level, the mRNA expression of *Cp*CDPK2A was the highest at 2 and 12 h during the *in vitro* infection of host cells by *C. parvum*. This is in agreement with previous reports of the gene expression (Mauzy et al., 2012; Lippuner et al., 2018). However, the addition of monoclonal or polyclonal antibodies against the enzyme to the *in vitro* culture of *C. parvum* did not block the invasion of the host cells, as antibodies might hardly block intracellular protein efficiency. This is expected if *Cp*CDPK2A plays only a secondary role in invasion intracellularly through its kinase activity. As one candidate inhibitor of *Cp*CDPK2A reduced parasite load in cell culture, it could be that the role of the enzyme in the life cycle is mostly on the growth of *C. parvum* after the invasion of host cells.

The identification of expression of *Cp*CDPK2A in both asexual and sexual stages indicates that it likely has multiple functions in *C. parvum* life cycle. In addition to its high expression in the invasive stages sporozoites and merozoites and in other asexual stages, *Cp*CDPK2A is one of the first *Cp*CDPKs that has been identified with high expression in macrogamonts. Previously, *Pf*CDPK1 was shown to be expressed in both male and female gametocytes of *Plasmodium falciparum*, and was important in male and female gametocyte development (Bansal et al., 2018). In contrast, *Pf*CDPK2 is essential in male gametocyte exflagellation but not essential in asexual development (Bansal et al., 2017). Therefore, *Cp*CDPK2A could play roles in both asexual and sexual development of *C. parvum*.

The expression of *Cp*CDPK2A in multiple developmental stages and its diverse functions in life cycle suggest that it is likely a good target for the development of drugs against *Cryptosporidium* spp. Indeed, the molecular docking of *Cp*CDPK2A in the present study has identified several candidate inhibitors of the enzyme. Among them, one, K292-0423, displayed the ability to inhibit the enzyme activity of *Cp*CDPK2A as well as significant *in vitro* anti-cryptosporidial activity at the micromole level. In recent years, several bumped kinase inhibitors of *Cp*CDPK1 have become the leading candidates for the development of novel treatment of cryptosporidiosis (Van Voorhis et al., 2021). Previously, using a similar approach, we also identified a *Cp*CDPK1 inhibitor (F083-0116) with inhibitory activities on the enzyme and *C. parvum* growth (Su et al., 2022). Thus, *Cp*CDPK2A might be another valid target for the development of treatment of cryptosporidiosis.

## Conclusion

In summary, results of the study suggest that *Cp*CDPK2A is highly expressed in diverse stages of *C. parvum* and could play some roles in the growth of the pathogen. As a result, inhibition of the enzyme by a candidate inhibitor can reduce the parasite load in *in vitro* culture. Nevertheless, more advanced studies using the newly developed CRISPR/Cas9 and cultivation tools should be conducted to elucidate the biological functions and action mechanism of the enzyme. This could lead to improved understanding of the biology of *Cryptosporidium* spp. and the development of effective therapeutics against cryptosporidiosis.

## Data Availability Statement

The original contributions presented in the study are included in the article/supplementary material, further inquiries can be directed to the corresponding authors.

## Author Contributions

LX and NL conceived the study. FS, YL, WC, and XC performed the experiments and statistical analysis. YL and ZZ contributed to the molecular docking work. YG and YF provided technical assistance. FS, YL, LX, and NL wrote the manuscript. All authors contributed to the article and approved the submitted version.

## Funding

This work was supported in part by Guangdong Major Project of Basic and Applied Basic Research (2020B0301030007), National Natural Science Foundation of China (32150710530), Natural Science Foundation of Guangdong Province (2019A1515011979), 111 Project (D20008), and Innovation Team Project of Guangdong University (2019KCXTD001).

## Conflict of Interest

The authors declare that the research was conducted in the absence of any commercial or financial relationships that could be construed as a potential conflict of interest.

## Publisher’s Note

All claims expressed in this article are solely those of the authors and do not necessarily represent those of their affiliated organizations, or those of the publisher, the editors and the reviewers. Any product that may be evaluated in this article, or claim that may be made by its manufacturer, is not guaranteed or endorsed by the publisher.

## Footnotes

^1^ http://cryptodb.org

^2^ https://myhits.sib.swiss/cgi-bin/motif_scan

^3^ http://www.megasoftware.net

^4^ https://imagej.nih.gov/ij/
